# Tuning of Ranvier node and internode properties in myelinated axons to adjust action potential timing

**DOI:** 10.1038/ncomms9073

**Published:** 2015-08-25

**Authors:** Marc C. Ford, Olga Alexandrova, Lee Cossell, Annette Stange-Marten, James Sinclair, Conny Kopp-Scheinpflug, Michael Pecka, David Attwell, Benedikt Grothe

**Affiliations:** 1 Division of Neurobiology, Department Biology II, Ludwig-Maximilians-Universität Munich, Munich D-82152, Germany; 2 Department of Neuroscience, Physiology and Pharmacology, University College London, London WC1E 6BT, UK

## Abstract

Action potential timing is fundamental to information processing; however, its determinants are not fully understood. Here we report unexpected structural specializations in the Ranvier nodes and internodes of auditory brainstem axons involved in sound localization. Myelination properties deviated significantly from the traditionally assumed structure. Axons responding best to low-frequency sounds had a larger diameter than high-frequency axons but, surprisingly, shorter internodes. Simulations predicted that this geometry helps to adjust the conduction velocity and timing of action potentials within the circuit. Electrophysiological recordings *in vitro* and *in vivo* confirmed higher conduction velocities in low-frequency axons. Moreover, internode length decreased and Ranvier node diameter increased progressively along the distal axon segments, which simulations show was essential to ensure precisely timed depolarization of the giant calyx of Held presynaptic terminal. Thus, individual anatomical parameters of myelinated axons can be tuned to optimize pathways involved in temporal processing.

## Abstract (web summary)

Action potential timing is fundamental to information processing, but its determinants are not fully understood. Here the authors demonstrate unexpected structural specializations of myelinated axons in the auditory brainstem that help to adjust action potential arrival time for sound localization.

## Graphical abstract

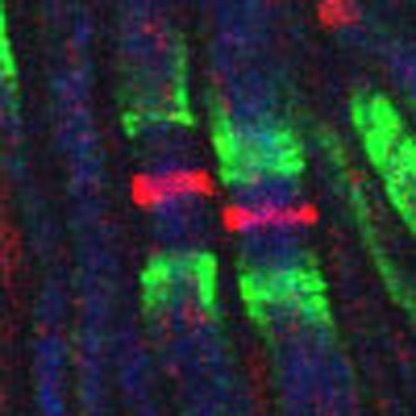

In the grey matter of the brain, neuronal dendrites and synapses have highly varying morphologies, which are adapted to particular information-processing tasks^1^. In contrast, the white matter is generally assumed to be comprised of axons with a canonical structure defined by a ratio of internode length (*L*) to myelin outer diameter (*D*) of ∼100 (refs 2, 3) and a ‘*g*-ratio' (the ratio of internodal axon diameter, *d*, to myelin diameter, *D*) of ∼0.75 (ref. 4). These concepts came from a theoretical analysis (tested against experimental data)^2^, which assumed that the physical size of all parts of myelinated axons should scale together, so that *L*/*D* should be constant (empirically measured as ∼100 for the peripheral nervous system), as should *g*=*d*/*D*; furthermore, a *g*-ratio of ∼0.6 was suggested to maximize the spread of signals between nodes (but the experimental value is 0.7–0.8, both in the peripheral nervous system and central nervous system (CNS)).

However, it would be surprising if myelinated CNS axons were as structurally invariant as is commonly assumed because the shape and timing of action potentials (APs) play a crucial role in synaptic transmission, information processing, rhythm generation and plasticity^5^,^6^,^7^,^8^,^9^, and variation of any of the geometrical parameters of myelinated axons could be used to tune their conduction speed and AP shape. For example, varying axon diameter at the node of Ranvier will alter the axial resistance and membrane capacitance (and possibly the number of voltage-gated Na^+^ channels) at the node, varying internodal axon diameter will change axial current flow along the axon, while altering internode length will change the fraction of the axon that benefits from the capacitance-reducing effect of myelination. Indeed, in the peripheral nervous system, the internode length is reduced near synaptic terminals (at the neuromuscular junction) to increase the effective Na^+^ current available to depolarize the terminals^10^, while in the CNS the myelination pattern can vary significantly along axons and between similar neurons, possibly to tune conduction time^11^,^12^,^13^,^14^. We demonstrate here how gradation of the node and internode properties along axons that are myelinated everywhere can tune conduction speed.

A model system for a detailed morphometric analysis of single myelinated axons in vertebrates is the sound localization circuitry that processes spatial information with exquisite precision. For instance, it has been shown that, in the bird pathway processing interaural time differences (ITD), variation in axon morphology is used for adjusting AP arrival times. There, thicker axons with longer internodes compensate for different axonal lengths^13^,^14^. The analogous mammalian auditory brainstem circuits that process ITDs and interaural level differences (ILDs) have similar exceptional needs for temporal precision in AP propagation, synaptic transmission and postsynaptic integration^15^. Both the ITD- and ILD-processing circuits contain a structure not present in birds, the medial nucleus of the trapezoid body (MNTB), which receives excitatory input from the contralateral cochlear nucleus globular bushy cells (GBCs) via the giant calyx of Held synapses. MNTB neurons are themselves glycinergic and provide inhibition to the binaural comparator neurons in the medial and lateral superior olive (MSO and LSO, respectively). While excitatory inputs from spherical bushy cells (SBCs) in the cochlear nucleus innervate MSO and LSO neurons directly (Fig. 1a), the inhibitory pathway includes the calyx of Held in the MNTB as an additional synapse. Despite the additional synaptic delay of several hundred microseconds, recent experimental and theoretical reports indicate an earlier arrival time for inhibitory than for excitatory input at the MSO^16^,^17^. Moreover, inhibitory and excitatory inputs arrive at about the same time at the LSO (reviewed in ref. 18), despite the additional synapse in the MNTB and the longer length of the inhibitory pathway. This suggests the involvement of structural adaptations for speeding up the conduction of inhibitory information in the binaural circuit. Indeed, it is reported that GBC axons represent the largest diameter fibres of the trapezoid body^18^,^19^. However, detailed knowledge of the geometrical parameters determining conduction velocity in GBC and SBC axons is lacking. Moreover, APs in the GBC–MNTB pathway are known to be particularly short^20^,^21^, can propagate along axons at extremely high frequencies (>600 Hz (refs 20, 22)) and are transmitted via the calyx of Held securely with short synaptic delays^23^. The influence of axon morphology on these properties has not been studied so far.

Here we show that GBC-myelinated axons, and in particular those of cells tuned to low sound frequencies (for which ILDs are minimal, and processing ITDs of only microseconds is important), deviate significantly from the canonically assumed structure in a paradoxical way: low-frequency (GBClat) fibres are thicker but exhibit a shorter internodal length than high-frequency (GBCmed) fibres. Our simulations as well as our recordings *in vitro* and *in vivo* indicate that this makes AP conduction particularly fast and precise. Furthermore, while it has been proposed that a long (20–40 μm) heminode is necessary for depolarizing the giant calyx of Held presynaptic terminal in juvenile animals^24^, we found that the heminode at the adult calyx is 10-fold shorter than this. Resolving this discrepancy, we demonstrate a novel mechanism that promotes calyx depolarization: the internode length decreases and the node diameter increases progressively towards the presynaptic terminal, and our detailed simulations predict that these gradations are crucial for precisely timed depolarization of the calyx of Held.

## Results

### Structural differences between SBC and GBC fibres

**Figure 1:**
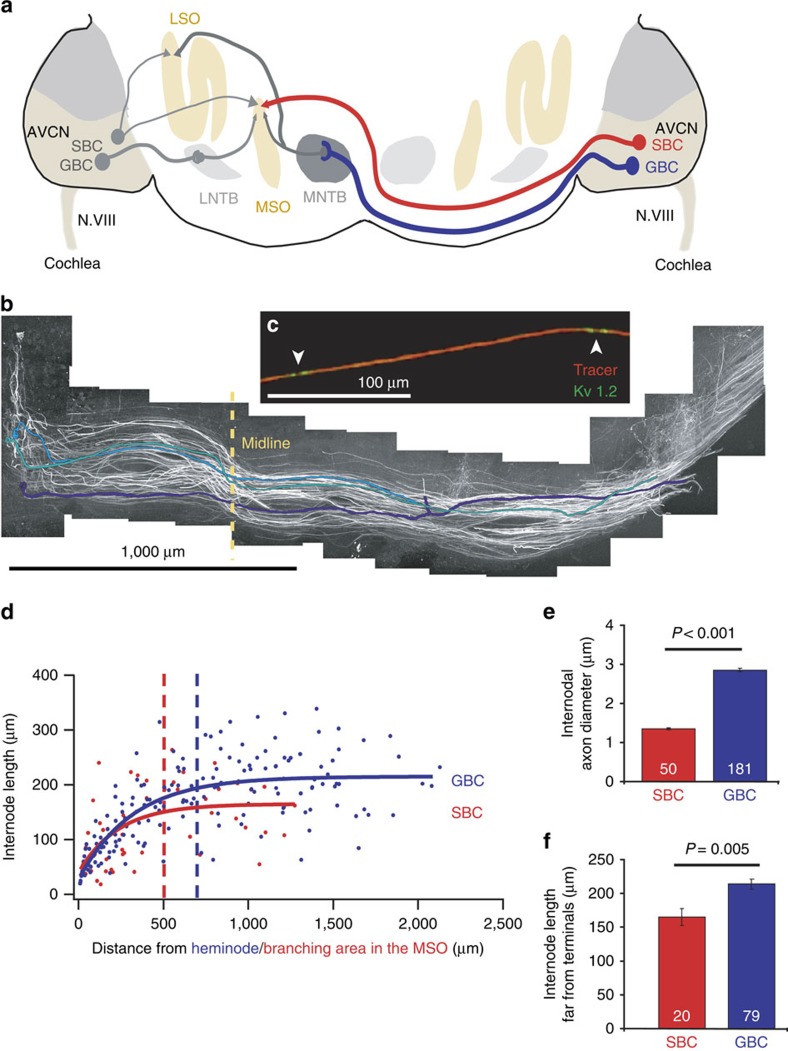
Afferent inputs to coincidence detection neurons of the MSO and LSO. (**a**) Simplified schematic of the mammalian ITD- and ILD-detection circuit. (**b**) Aligned projections of confocal image stacks from transverse brainstem slices at the level of the trapezoid body. GBC axons were anterogradely traced with tetramethylrhodamine dextran. Three individual fibres are highlighted in colour. (**c**) Magnification of a GBC axon segment (red) including two nodes of Ranvier (arrowheads). The position of juxtaparanodal immunolabelled K_v_1.2 channels is shown in green. (**d**) Internode length in GBC and SBC axons plotted against the distance from the heminode and the branching area in the MSO, respectively. (**e**) The mean internodal axon diameter is significantly larger in GBC axons than in SBC axons (*P*<0.001; Mann–Whitney test). (**f**) The mean internode length is larger in GBC axons than in SBC axons (*P*=0.005, *t*-test). For GBC and SBC axons, the mean INL was calculated from internodes located >700 μm away from the heminode and from internodes located >500 μm away from the MSO, respectively (dashed lines in **d**). Numbers on bars are of internodes in 17 GBC and 7 SBC axons. Data in **e**,**f** are represented as mean±s.e.m. Scale bars, 1,000 μm (**b**) and 100 μm (**c**).

We first measured the internodal axon diameter and internode length in SBC fibres innervating the MSO and in GBC fibres innervating the MNTB. A combination of dye filling of axons (Fig. 1b) and immunohistochemical labelling of nodal and juxtaparanodal proteins allowed us to measure axon diameter, to unambiguously identify the position of nodes of Ranvier along axons (Fig. 1c) and to follow single axons with an identified termination site for up to 2,200 μm. The internodal axon diameter of GBC axons was more than twice that of SBC axons (measured far from their synaptic terminals where internode length is constant (Fig. 1d): SBC 1.35±0.03 μm at >500 μm from the medial somatodendritic region of the MSO, GBC 2.87±0.05 μm at >700 μm from the terminals; *P*<0.001; Mann–Whitney test; Fig. 1e). The mean internode length (far from the synaptic terminals) in the thicker GBC fibres was also larger than in the thinner SBC fibres (SBC 164.8±12.4 μm, GBC 208.9±7.0 μm, *P*=0.005, *t*-test; Fig. 1f). Simulations presented below assess how this difference of parameters affects the conduction speed in the pathways bringing excitatory and inhibitory inputs to the MSO.

### GBC fibre parameters vary with tonotopic identity

**Figure 2:**
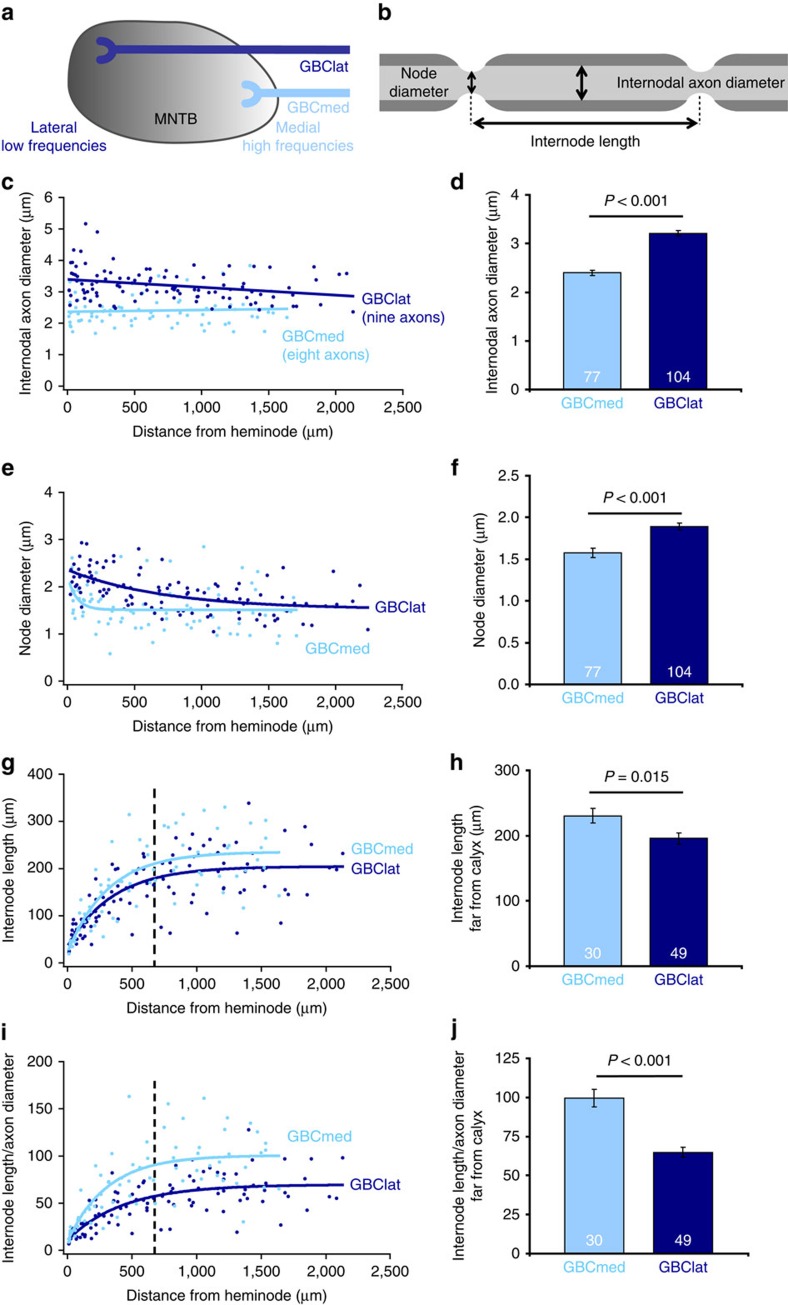
Structural parameters of GBC axons vary systematically with their termination region in the MNTB and, hence, their tonotopic identity. (**a**) Schematic of the tonotopically organized MNTB with GBC axons terminating in the lateral low-frequency region (GBClat; dark blue) and in the medial high-frequency region (GBCmed; light blue). (**b**) Schematic of a myelinated axon segment illustrating the structural parameters analysed. (**c**,**e**,**g**) Internodal axon diameter, node diameter and internode length plotted as functions of distance from the heminode. In GBCmed and GBClat axons, node diameter increases (**e**), whereas internode length decreases (**g**) closer to the synaptic terminals (see also Supplementary Fig. 1). The *x*-value of each data point represents the position of the centre of each internode or node along the axon. (**d**,**f**) Internodal axon diameter and node diameter are larger in GBClat than in GBCmed axons (averaged over all positions; *P*<0.001 for internodal axon diameter, Mann–Whitney test; *P*<0.001 for node of Ranvier diameters, *t*-test). (**h**) For >700 μm upstream of the heminode (dashed line in **g**) internode length is significantly larger in GBCmed than in GBClat axons (*P*=0.015, *t*-test). (**i**) *L*/*d* ratio in GBCmed and GBClat axons plotted against distance from heminode. (**j**) For internodes >700 μm away from the heminode (dashed line in **i**), *L*/*d* ratio is larger in GBCmed than in GBClat axons (*P*<0.001, Mann–Whitney test). Numbers on bars are of internodes or nodes in nine GBClat and eight GBCmed axons. Data in **d**,**f**,**h**,**j** are represented as mean±s.e.m.

The MNTB is a tonotopically organized nucleus, with GBC axons conveying high sound frequencies targeting the medial region and those conveying low sound frequencies targeting the lateral region^25^ (Fig. 2a). Tracing individual GBC axons to their calyx of Held terminals enabled us to categorize them by their termination region in the MNTB, that is, providing input to the medial half of the nucleus (GBCmed or high-frequency) or to the lateral half (GBClat or low-frequency). In nine GBClat and eight GBCmed axons we determined internodal axon diameter, node of Ranvier diameter and internode length, three major structural determinants of the conduction speed of APs^26^, at different points along the axon (Fig. 2b). Interestingly, we observed systematic differences in the two populations of GBC axons. GBClat axons had a significantly larger mean internodal axon diameter than GBCmed axons (GBClat 3.21±0.05 μm averaged over all positions, GBCmed 2.40±0.05 μm, *P*<0.001, Mann–Whitney test; Fig. 2b–d). The diameter of nodes of Ranvier was also larger in GBClat axons than in GBCmed axons (GBClat 1.91±0.04 μm, GBCmed 1.60±0.05 μm, *P*<0.001, *t*-test; Fig. 2e,f). However, despite the larger diameter of GBClat axons, the mean length of internodes located further than 700 μm away from the calyx (where internode length was constant, see below) was smaller in GBClat than in GBCmed axons (GBClat 195.7±8.5 μm, GBCmed 230.4±11.1 μm, *P*=0.015, *t*-test; Fig. 2g,h). This surprising finding contradicts the long-assumed notions^2^ that internode length is proportional to axon diameter, and that axons with similar function exhibit structural similarity.

**Figure 3:**
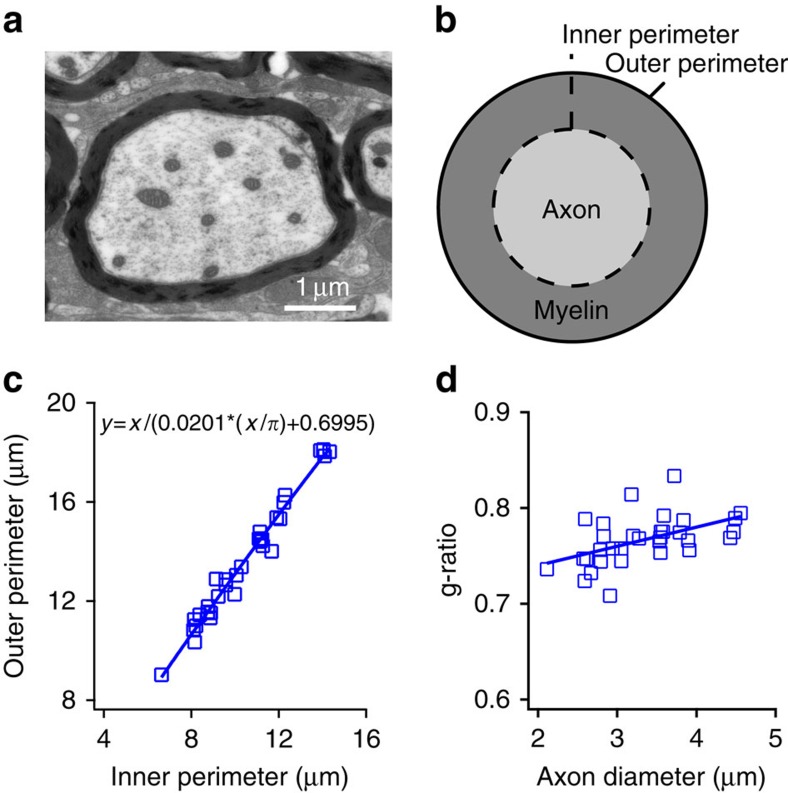
Myelin thickness in GBC fibres. (**a**) Transmission electron micrograph of a GBC axon. (**b**) Schematic cross-section of a myelinated axon illustrating the measured parameters. (**c**) Outer (fibre) perimeter plotted against inner (axon) perimeter. For calculating *g*-ratios, perimeter measurements were preferred to diameter measurements because the latter can lead to quite variable results, depending on where diameters are measured. (**d**) *g*-ratios (inner perimeter/outer perimeter) from 32 individual fibres plotted as a function of axon diameter. *g*-ratio values were estimated for the mean internodal axon diameters of GBCmed and GBClat axons (0.748 and 0.764) by interpolating using the line of best fit. Scale bar, 1 μm (**a**).

This structural difference became even more apparent when the ratio of internode length to internodal axon diameter (*L*/*d*) was compared (Fig. 2i). GBCmed axons had, far from the calyx, a mean *L*/*d* ratio of ∼100. In contrast, for GBClat axons the mean ratio was ∼35% smaller (GBClat 64.9±3.1; GBCmed 99.6±5.7; *P*<0.001, Mann–Whitney test; Fig. 2j). Both of these values are lower than the canonical value of 133, which is predicted (for a *g*-ratio of 0.75, see Fig. 3d below and ref. 4) from Rushton's^2^ classical summary of the properties of peripheral nervous system fibres as having a ratio of internode length to myelin outer diameter of ∼100. These data suggest strongly that there are systematic differences in the structure of GBC axons that can be related to the tonotopic organization, and thus to the function of these axons in the sound localization circuits processing interaural time and level differences.

### Myelin thickness in GBC fibres

Another major structural determinant of AP conduction velocity in myelinated axons is the thickness of the myelin sheath^26^. Increasing the number of myelin wraps lowers the capacitance along the internode, reducing the time required to charge the internodal membrane. This means that consecutive nodes of Ranvier reach threshold quicker and, therefore, the conduction velocity is increased. In addition, it increases the effective membrane resistance, thus reducing radial current leak between consecutive nodes of Ranvier. We determined myelin thickness from transmission electron micrographs of GBC fibres (Fig. 3a) in parasagittal ultrathin sections by measuring the axon perimeter and the fibre perimeter (Fig. 3b,c). The *g*-ratio was calculated by dividing the measured inner (axon) perimeter by the measured outer (myelin) perimeter^27^. As expected^28^, *g*-ratio increased slightly with axon diameter, that is, myelin thickness relative to axon diameter decreased at larger axon diameters (Fig. 3d). Estimated *g*-ratio values for the mean internodal axon diameter of GBCmed and GBClat axons were 0.748 and 0.764, respectively, which were used in our detailed computational axon model described below.

### Spatial variation of axon structure towards the calyx

In addition to the variation of the mean geometrical parameters between fibres with different tonotopic identity, we found a systematic variation of structural parameters along the distal part of all GBC fibres (Fig. 2e,g). From ∼700 μm before reaching the heminode, the internode length decreased progressively from 195.7±8.5 to 53.5±0.2 μm at the last internode in GBClat fibres, and from 230.4±11.1 to 36.9±3.0 μm at the last internode in GBCmed fibres (Fig. 2g and Supplementary Fig. 1A). The observed decrease in internode lengths was not a secondary effect necessary to allow fibre branching^29^,^30^, as only 5 of the 17 analysed GBC axons gave rise to a branch in their distal part. A gradation along the axon was also found for node diameters (Fig. 2e and Supplementary Fig. 1B). On average, node diameter increased by 35%, approaching the synaptic terminal in the most distally located eight nodes of Ranvier of GBClat fibres, and by 40% in the most distally located four nodes of GBCmed fibres. This increase in node diameter will decrease the nodal axial resistance and (for a fixed channel density per membrane area) increase the number of voltage-gated Na^+^ channels per node which, together with the increased density of nodes associated with shorter internode lengths, could support an increased current flow down the axon to promote reliable AP invasion into the giant calyx of Held. We test this idea in simulations presented below.

### Morphometry of the pre-terminal axon segment

**Figure 4:**
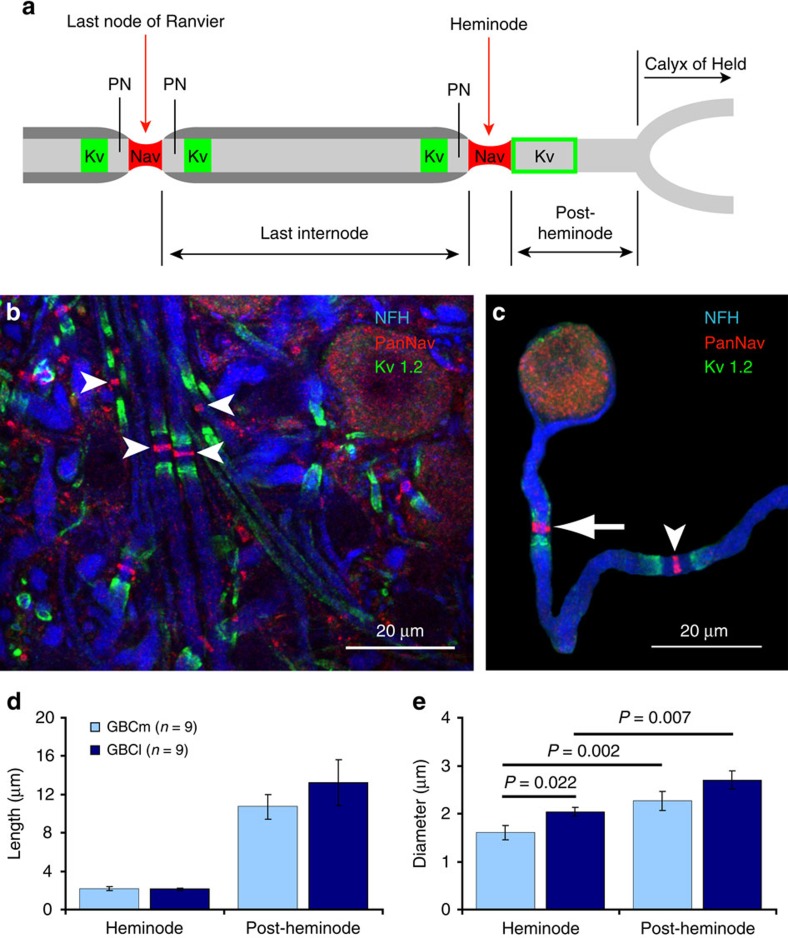
Morphometry of the pre-terminal segment of GBC axons. (**a**) Schematic of the terminal part of a GBC axon (grey) illustrating the measured parameters. Red segments indicate the position of sodium channels at the last node of Ranvier and the heminode. PN denotes paranode. Green rectangles indicate the position of juxtaparanodal K_v_1.2 channels and the green open box indicates the position of pre-calyceal K_v_1.2 channels. (**b**) Maximum-intensity projection of confocal images displaying axons (blue, neurofilament heavy chain) in the MNTB. Arrowheads indicate nodes of Ranvier stained with an anti-pan-Na_v_ antibody (red). K_v_1.2 channels are shown in green. (**c**) Distal part of a GBC axon (blue) after digital extraction from the surrounding image. The last node of Ranvier (arrowhead) and the heminode (arrow) are labelled with an anti-pan-Na_v_ antibody (red). Juxtaparanodal and pre-calyceal K_v_1.2 channels are shown in green. (**d**) Heminode length and post-heminode length do not differ between GBCmed and GBClat axons. (**e**) The heminode diameter is larger in GBClat than in GBCmed axons (*P*=0.022, Mann–Whitney test). In GBCmed and GBClat axons the post-heminode diameter is larger than that of the heminode (*P*=0.002 for GBCmed, paired *t*-test; *P*=0.007 for GBClat, paired *t*-test). Data in **d**,**e** are represented as mean±s.e.m. Scale bars, 20 μm (**b**,**c**).

The myelinated part of GBC fibres was followed by a short unmyelinated^24^ pre-terminal axon segment that connected the last internode to the calyx of Held. Immunohistochemical staining revealed that it consisted of a sodium channel-containing heminode, directly adjacent to the last internode, and a sodium channel-free segment, which we refer to as the post-heminode (Fig. 4a–c). The heminode had a similar length in medially and laterally terminating fibres (2.13±0.13 μm in GBClat and 2.18±0.19 μm in GBCmed; Fig. 4d). In accordance with the differences in internodal axon and node diameters in GBClat and GBCmed fibres (Fig. 2c–f), the heminode diameter was significantly larger in GBClat fibres (GBClat 2.04±0.09 μm, GBCmed 1.60±0.15 μm, *P*=0.022, Mann–Whitney test, Fig. 4e). The diameter of the post-heminode was significantly larger than the heminode diameter in both GBClat (2.70±0.19 μm, *P*=0.007, paired *t*-test) and GBCmed axons (2.26±0.20 μm, *P*=0.002, paired *t*-test). The post-heminode ranged in length from 7 to 30 μm (mean 13.2±2.1 μm) in GBClat, and from 4 to 14 μm (10.7±1.3 μm) in GBCmed fibres (Fig. 4d). Consequently, it constitutes a large capacitance and has potentially strong effects on AP invasion into the calyx of Held.

### Dependence of simulated conduction velocity on *L*/*d* ratio

**Figure 5:**
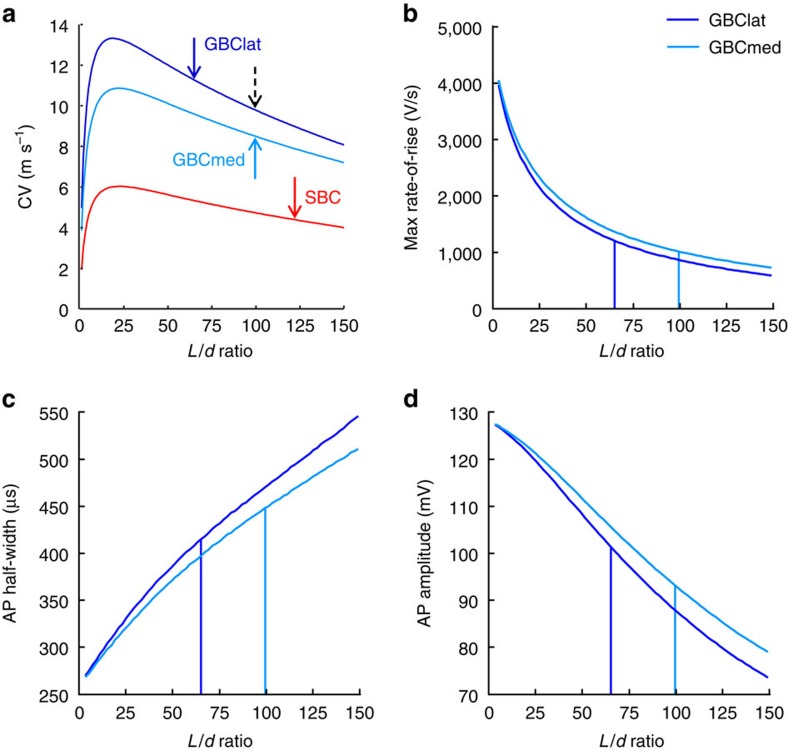
Computer simulations exploring the effect of morphological parameters on action potential propagation in SBC and GBC axons. (**a**) Dependence of conduction velocity on *L*/*d* ratio in GBClat, GBCmed and SBC axons. For GBClat, GBCmed and SBC axons the internodal diameters were fixed at 3.06, 2.40 and 1.35 μm, respectively, and the nodal diameters were fixed at 1.68, 1.50 and 0.85 μm. At low *L*/*d* ratios the conduction velocity decreases because a smaller fraction of the axon is myelinated, while at long internode lengths it decreases because of inefficient transfer of depolarization between nodes. Solid arrows indicate the experimentally determined *L*/*d* ratios. Dashed arrow shows extra simulated *L*/*d* ratio for GBClat. (**b**–**d**) Dependence of the maximum rate of rise (**b**), half-width (**c**) and amplitude (**d**) of the simulated axonal AP on *L*/*d* ratios in GBCmed and GBClat fibres. Vertical lines indicate the experimentally observed *L*/*d* ratios in GBCmed and GBClat fibres.

To explore how the morphological differences between SBC, GBClat and GBCmed axons affect AP propagation, we carried out detailed computer simulations^31^ informed by the measured anatomical parameters for each axon type, as described in the Methods section and Supplementary Tables. The conduction velocity of the computed AP far from the synaptic terminals was 4.4 m s^−1^ in SBC axons, 8.5 m s^−1^ in GBCmed axons and 11.3 m s^−1^ (33% faster) in GBClat axons that have a lower ratio of internode length to axon diameter (arrowed values in Fig. 5a). The lower velocity in SBC axons than in GBC(lat) axons correlates well with inhibitory information arriving before excitatory information at the MSO^17^ (Fig. 1a).

To estimate how much of the 33% increase in conduction velocity in GBClat axons (compared with GBCmed axons) was due to their smaller internode length to axon diameter (*L*/*d*) ratio, we systematically explored the effect of varying the internode length (without changing the axon diameter, node diameter or *g*-ratio) in both populations of axon (Fig. 5a). At low internode lengths the conduction velocity decreases because a smaller fraction of the axon is myelinated, while at long internode lengths it decreases because of inefficient transfer of depolarization between nodes, giving an optimum value of *L*/*d* to maximize conduction velocity^32^. Increasing the internode length to increase *L*/*d* in GBClat axons from 64.9 to 99.6, the ratio we found in GBCmed axons (dashed arrow in Fig. 5a), decreased the conduction speed from 11.3 to 9.8 m s^−1^. Thus, the smaller *L*/*d* ratio in GBClat fibres, which generates a conduction speed closer to the highest value that can be produced for these axons by varying *L*/*d* in our simulations (Fig. 5a), increases the conduction velocity by 15% (while 18% of the increase in speed of these axons is due to the larger internode and node diameters).

Both lateral and medial GBC axons have an internode length greater than the value that maximizes conduction velocity in our simulations (Fig. 5a), which has been suggested to decrease energy use with little decrease in conduction velocity^2^. We assessed this idea by calculating (from our simulations) the Na^+^ entering to generate an AP for internode length to axon diameter ratios in Fig. 5a chosen to be either as observed experimentally (solid arrows in Fig. 5a) or which would maximize the conduction speed (peaks of the curves in Fig. 5a). For SBC, GBCmed and GBClat axons, the observed values of *L*/*d* reduced the conduction speed by 28%, 22% and 15%, respectively (compared with the maximum velocities attainable in our simulations) but reduced the energy needed for Na^+^ pumping by a larger percentage (53%, 49% and 45% respectively).

### Dependence of simulated AP shape on *L*/*d* ratio

The presynaptic AP waveform is a crucial determinant of the timing (synaptic delay) and strength of synaptic transmission^7^,^33^. In sound localization circuits, brief APs are not only crucial for keeping synaptic delays short and for preserving temporal information during synaptic transmission but also support precise and reliable propagation of high-frequency AP trains up to 500–800 Hz as observed *in vivo*^34^,^35^, a prerequisite for neurons tuned to low sound frequencies to respond in a cycle by cycle manner to a specific phase of the sound wave^36^. The simulated AP in GBClat fibres exhibited a more rapid rate of rise of the upstroke (1,202 V s^−1^), a shorter half-width (0.41 ms) and larger amplitude (101 mV) than in GBCmed fibres (rate of rise 1,039 V s^−1^; half-width 0.44 ms; amplitude 94.1 mV; Fig. 5b–d). Notably, this effect depended on the smaller *L*/*d* ratio in GBClat fibres because increasing the ratio to 99.6 decreased the rate of rise by 29% to 859 V s^−1^, increased the AP half-width by 14% to 0.47 ms and decreased the amplitude by 13% to 87.8 mV. This effect of internode length probably occurs because, for larger *L*/*d* ratios, less efficient transfer of current from one node to an adjacent node results in less synchronous activation of Na^+^ channels (resulting in a less rapid rate of rise) and to a less rapid and synchronous activation of potassium channels, both of which may contribute to an increase in half-width. Briefer APs in GBClat fibres should allow for shorter intervals between APs and hence temporally more reliable responses to fast incoming signals (that is, allowing the phase locking that is exhibited by low-frequency GBC fibres)^36^, and a larger amplitude may increase the reliability (by increasing the safety factor) of AP propagation in these axons. Moreover, briefer APs have been shown to be associated with a smaller synaptic delay^7^,^33^,^37^, and increased fidelity of synaptic transmission^20^, which is fundamental to binaural processing of temporal information in sound localization circuits^15^,^38^.

### Factors aiding AP invasion into the calyx

**Figure 6:**
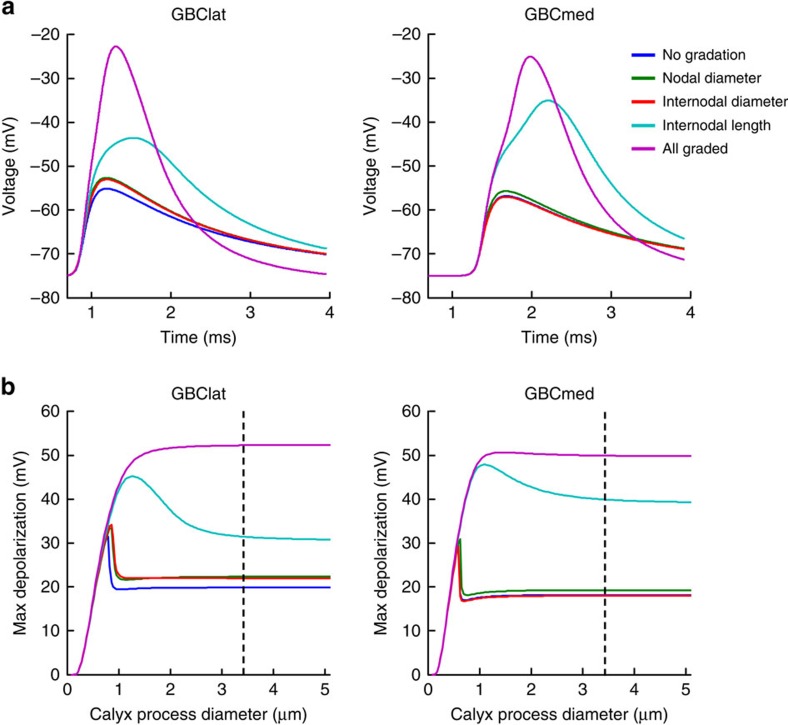
Computer simulations exploring the effect of morphological parameters on action potential invasion into the calyx of Held. (**a**) Propagation of APs into the GBClat (left) and GBCmed (right) axon terminals is facilitated by including experimentally observed gradations of the axon and myelin parameters. The membrane potential waveform in the calyx has a shorter delay, larger amplitude and faster rate of rise when gradations of node diameter, internode diameter and internode length are included. (**b**) Action potential propagation into the calyx of GBClat (left) and GBCmed (right) axons occurs over a wider range of calyx geometries when experimentally observed gradations of the axon anatomy are included. When the diameter of the calyx processes was varied, while retaining the same calyx membrane surface area (thus, altering the longitudinal resistance into the calyx processes, but retaining the same conductance and capacitance across the calyx membrane), it was found that graded properties of the axon towards the end of the presynaptic axon allowed propagation of the action potential into the synaptic terminal over a wider range of calyx dimensions. Dotted line indicates calyx process diameter for which the membrane potential waveforms are shown in **a**. Calyx surface area was 1,700 μm^2^ for GBClat and 1,250 μm^2^ for GBCmed.

To test the effect of the internode length and node diameter gradations towards the end of GBClat axons on AP invasion into the calyx of Held, we simulated the end terminal as three cylinders corresponding to the heminode, post-heminode and calyx (Fig. 4a, details of the parameters chosen are in the Methods section and Supplementary Tables). The dimensions of the heminode and post-heminode were taken from the anatomical observations above. The calyx of Held was modelled as an equivalent cylinder that represented the complex processes that make up the calyx (see Methods section) and had a total surface area of 1,700 μm^2^ (in the observed range^39^). Without any spatial gradation of internode length, node diameter and internode diameter with distance from the synaptic terminal, for GBClat axons APs did not propagate successfully into the calyx (the maximum depolarization was 19.9 mV, measured at the distal end of the calyx, Fig. 6a dark blue trace). After introducing the observed spatial gradation of internode length (shorter nearer the calyx), AP propagation into the calyx was facilitated (the maximum depolarization was 31.4 mV, Fig. 6a light blue trace). A facilitation was achieved by introducing only the gradation of internode length, without introducing the gradation of internode and node diameters. However, additional introduction of the observed gradation of node diameter and internode diameter led to a more rapid entry of the AP into the calyx (a 34-μs speeding of the AP was observed with gradation of internode length only versus a 51-μs speeding for all gradations included; delays were measured from the time for the AP to pass −60 mV). The gradation of node diameter and internode diameter, when introduced alone, had little effect on the AP waveforms (Fig. 6a, green and red traces). However, when the gradation of node diameter and internode diameter was introduced on top of that of the internode length, this led to a more rapid rate of rise of the AP upstroke (maximum rate of rise: 96.7 V s^−1^ without any gradations, 126.0 V s^−1^ with gradation of internode length only and 155.7 V s^−1^ with gradations of node diameter, internode diameter and internode length) and a larger AP amplitude (52.3 mV, Fig. 6a magenta trace).

The calyx shape varies considerably during development^40^. We varied systematically the diameter of the processes of the simplified calyx while keeping the total calyx surface area constant (by adjusting the process length) to examine how robustly the AP invaded calyces of different shapes, and the role of the gradation of axonal properties in ensuring this invasion. The simulations revealed that gradations of internode length, and of nodal and internodal diameters, are essential for successful propagation of the AP into the GBClat terminal over a wide range of calyx geometries (Fig. 6b), as observed experimentally^20^. Similar behaviour was observed in simulations of GBCmed axons (Fig. 6a,b), except that the surface area of the calyx had to be reduced to 1,250 μm^2^ (also in the observed range^39^) in order for APs to depolarize the calyx beyond −40 mV. For both panels in Fig. 6b, the initial rise in the maximum voltage as the process diameters are increased from zero is due to the resulting decrease in axial resistance along the calyx. Since we are measuring the voltage at the distal end of the calyx, a lower axial resistance leads to less voltage drop along the calyx. However, for all the curves, except those in which all the parameters are graded with position, as the diameter of the processes increases further the calyx becomes more isopotential and the effective input resistance at the proximal end of the calyx decreases. This decrease in input resistance reduces the depolarization occurring in the heminode and post-heminode and therefore prevents activation of voltage-gated Na^+^ channels in the heminode. Thus, a decrease in the maximum depolarization in the calyx occurs at the point when the threshold for activation of Na channels in the heminode is no longer reached.

### Measurement of conduction speed in GBCmed and GBClat fibres

**Figure 7:**
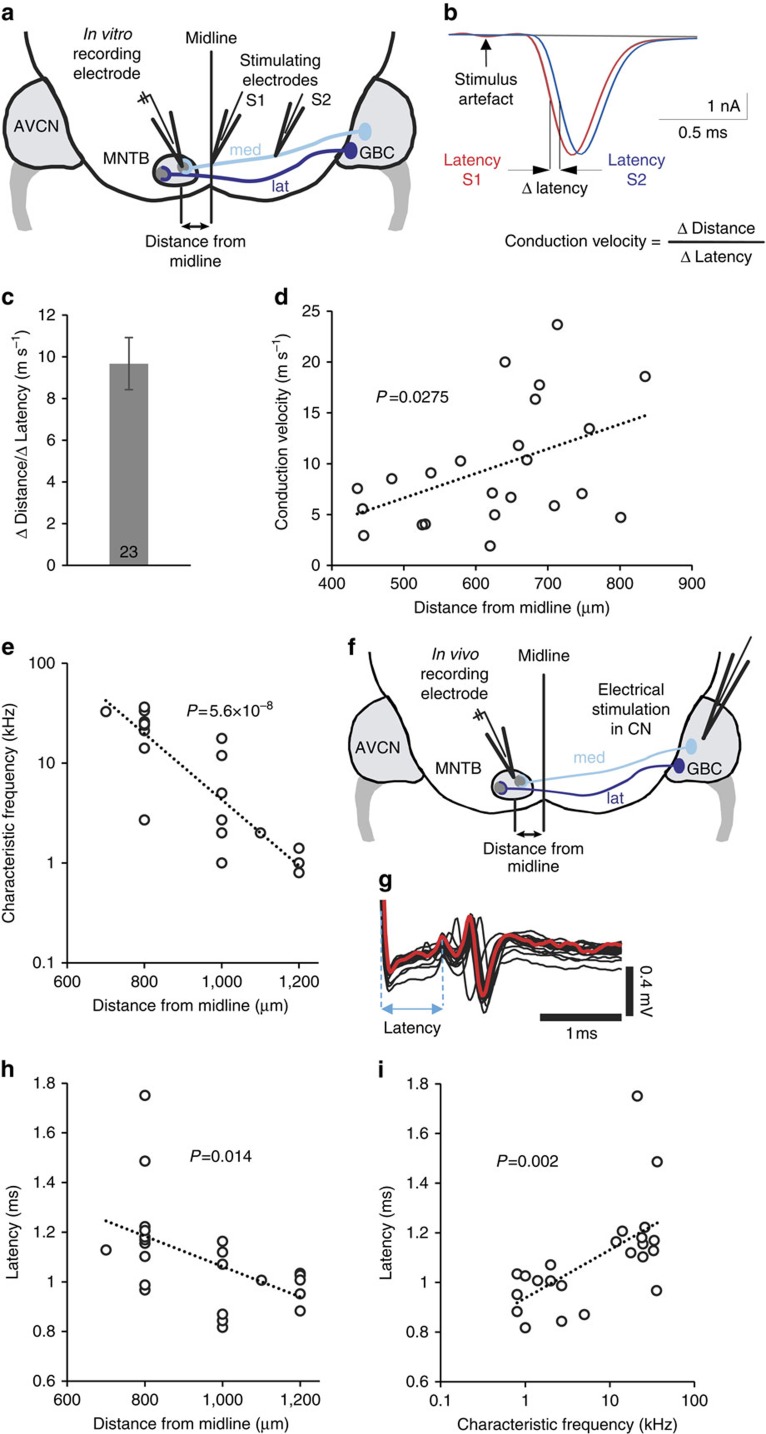
Electrophysiological measurements of axonal conduction velocities and neuronal response latencies along the tonotopic axis in the MNTB. (**a**) Schematic of a coronal brain slice illustrating the measurements of conduction velocity in GBC fibres *in vitro*. (**b**) Averaged EPSCs of an MNTB neuron in response to electrical stimulation of the afferent fibres at two locations as shown in **a**. Response latency was measured at half-maximal amplitude of the postsynaptic response. Axonal conduction speed was calculated by dividing the distance between the two stimulating electrodes by the difference in the latency of the postsynaptic response when stimulating with electrode S1 or S2. (**c**) The mean conduction velocity measured in GBC fibres far from the calyx (±s.e.m.). (**d**) Estimated conduction velocity of GBC fibres plotted against the mediolateral position of their termination region in the MNTB (slope is significantly greater than zero, *P*=0.0275 from F test). (**e**) Characteristic frequency of MNTB neurons recorded *in vivo* plotted against their mediolateral position (distance from midline). The slope is significantly greater than zero (*P*=5.6 × 10^−8^ from F test). (**f**) Schematic illustrating the latency measurements *in vivo*. (**g**) Example traces of MNTB principal cell responses to repeated electrical stimulation (10 μA). One trace is highlighted in red to illustrate how latency was measured in single traces. Electrical artefacts at the beginning of traces are truncated. (**h**) The mean latency of presynaptic responses to electrical stimulation (10–40 responses) from calyceal inputs plotted against the tonotopic location of the MNTB principal cells from which recordings were obtained (slope is significantly greater than zero, *P*=0.014 from F test). (**i**) The mean latency of presynaptic responses to electrical stimulation plotted against the postsynaptic MNTB neuron's characteristic frequency (slope is significantly greater than zero, *P*=0.002 from F test). Data in **c** are represented as mean±s.e.m.

To verify the simulated conduction velocities of GBC fibres we performed *in vitro* recordings in brain slices. The conduction velocity of single GBC fibres was measured far from the calyx of Held by whole-cell patch clamp recording from MNTB neurons while electrically stimulating the trapezoid body fibres in two locations (Fig. 7a). One stimulating electrode was placed near the midline and the other one further on the contralateral side. To estimate the axonal conduction velocity, the distance between the two stimulating electrodes was divided by the difference in the latency of the postsynaptic response when stimulating with electrode S1 or S2 (Fig. 7b). These experiments gave a mean axonal conduction speed for GBC axons of 9.67±1.25 m s^−1^ (*n*=23; Fig. 7c), which is comparable to the simulated conduction speed values (8.5 m s^−1^ in GBCmed and 11.3 m s^−1^ in GBClat axons, see Fig. 5a). The estimated conduction velocity tended to increase with distance from the midline (*P*=0.0275), that is, laterally terminating GBC fibres tended to have a higher conduction velocity than medially terminating GBC fibres (Fig. 7d), as in Fig. 5a. Thus, these results indicate that the parameters chosen for the computer simulations result in realistic conduction velocities, and successfully predict a greater conduction speed for GBClat than for GBCmed fibres.

To further investigate differences in conduction speed between high- and low-frequency fibres, we performed *in vivo* recordings extracellularly at different positions in the MNTB. First, responses of single MNTB neurons were elicited by acoustic stimulation and the characteristic frequency (CF) of each neuron was determined. Consistent with previous reports^25^,^41^, the CF correlated with the neuron's position along the mediolateral axis of the MNTB (*P*=5.6 × 10^−8^), with high-CF neurons being located in the medial part of the nucleus and low-CF neurons being located in the lateral part (Fig. 7e). After determining the neuron's CF, responses were evoked electrically in the cochlear nucleus, circumventing delays between the sensory periphery and the cochlear nucleus (Fig. 7f; histological verification of the position was obtained by DiI labelling). Only neurons with characteristic complex waveforms consisting of a prepotential (reflecting the activity of the calyx of Held presynaptic terminal) followed by a postsynaptic spike were included in the analysis (Fig. 7g). The latency of axonal AP propagation was defined as the time between the peak of the stimulation artefact and the peak of the calyceal AP (the peak of the prepotential of the complex waveform recorded extracellularly; Fig. 7g). The latency of the prepotential decreased with increasing distance of the recording site from the midline (*P*=0.014; Fig. 7h). Accordingly, the latency correlated positively with the neuron's CF (*P*=0.002; Fig. 7i). Thus, as in the brain slices, these results strongly indicate that AP conduction velocity is higher in low-frequency GBClat axons than in high-frequency GBCmed axons, as predicted in Fig. 5a.

## Discussion

We have identified several unusual morphological parameters of axons transmitting information to the MSO and LSO. First, the diameter and internode length of GBC axons, which transmit information that passes through an extra synapse in the MNTB and provide inhibition to the MSO and LSO (Fig. 1a), are larger than those of the SBC axons transmitting excitatory information. The resulting increase in transmission speed of the inhibitory information could explain why inhibitory information can arrive approximately simultaneously with the excitatory information in the LSO and even before excitatory information at the MSO^17^,^18^ despite the additional synaptic delay in the MNTB. An analysis of the analogous ITD-processing circuit in birds suggests that a different anatomical tuning mechanism occurs: differences of axon diameter and internode length may tune different length branches of the same myelinated axons to provide the required AP arrival times^13^,^14^.

Second, the present study provides evidence that structural specializations in myelinated GBC axons that strongly deviate from the canonically assumed behaviour can additionally tune their conduction velocity, and thus the timing of APs. Specifically, we found systematic differences in the structure of GBC axons that correlate with their target region in the MNTB, and hence with their tonotopic identity. Lateral (low frequency) GBCs had a larger internodal axon diameter (d), but, paradoxically, a shorter internode length (*L*) than medial (high frequency) GBCs. This result contradicts the principle of structural similarity of myelinated axons^2^, which suggested that *L*/*d* should be the same for all myelinated axons. Our simulations predict that this difference in structure results in GBClat axons conducting APs 33% faster than GBCmed axons (Fig. 5a), which may serve to compensate for the longer path length of GBClat axons and allow simultaneous arrival at the MNTB of afferent signals tuned to different sound frequencies. About half of this increase in speed in GBClat compared with GCBmed axons is attributable to the lower *L*/*d* ratio in GBClat fibres. In addition, the lower *L*/*d* ratio increased the rate of rise and decreased the half-width of the AP in GBClat axons, which is a prerequisite for temporally precise synaptic transmission at the calyx of Held. A first indication of differences of axon structure potentially related to tonotopic identity came from an investigation of peripheral auditory axons^42^. In the light of our analysis of how individual structural parameters contribute to myelinated axon function in the CNS, it is likely that these peripheral nervous system adaptations are also crucial for increasing the reliability and temporal precision of AP propagation in axons conveying low-frequency sound information for binaural processing.

It has been questioned whether the auditory circuits involved in temporal processing, which exhibit energy-demanding high-frequency APs, are designed to use energy efficiently^43^. Far from their synaptic terminals, SBC axons had a ratio of internode length to internodal axon diameter of 122, which is similar to the canonical value of 133 derived from peripheral axons^2^ by assuming a *g*-ratio of 0.75, while both GBC axon types had significantly lower values (GBCmed 99.6 and GBClat 64.9), which increases their conduction speed (Fig. 5a). It is noteworthy that the ratio of internode length to internodal axon diameter in GBC and SBC axons is significantly higher than the value that maximizes conduction speed (Fig. 5a)—a situation that our simulations show reduces energy consumption^2^ on pumping out the Na^+^ that enters to generate the AP—and that the value that maximizes speed is much less than the value of 133 expected from peripheral nerve data. For SBC, GBCmed and GBClat axons, the observed values of *L*/*d* reduced the conduction speed compared with the maximum velocities attainable, but reduced the energy needed for Na^+^ pumping by a larger percentage.

Finally, we found unexpected gradations of geometrical parameters in CNS axons of the GBC–MNTB pathway, which seem to be structurally and functionally analogous to those of peripheral nervous system motorneurons close to the neuromuscular junction. In GBCs the internode length, internodal axon diameter and node diameter all change systematically with distance from the synaptic terminal in the calyx of Held (Fig. 2). This spatial variation is crucial for ensuring a rapid depolarization of the calyx of Held (Fig. 6a), which is reliable over a range of simulated calyx morphologies (Fig. 6b). Strikingly, in our simulations, for large calyces the shortening of internode length near the synaptic terminal (which has previously been suggested to promote synaptic depolarization in the peripheral nervous system^10^ and has been speculated to also exist in GBC fibres to promote depolarization of the long unmyelinated pre-terminal axon segment^24^) was not sufficient to ensure that a large and rapid depolarization occurred in the synaptic terminal, but additionally including the spatial variation of nodal and internodal axon diameters did ensure this. Interestingly, it has been proposed that 20–40-μm-long Na_v_-positive structures in the MNTB of young rats represent heminodes of GBC fibres, and that these putative heminodes might be necessary for depolarizing the giant calyx of Held presynaptic terminal^24^. However, our labelling with Na_v_, neurofilament heavy chain (NFH) and K_v_1 antibodies showed that the heminode in pre-calyceal segments of GBC axons of adult gerbils is only ∼2 μm long. This indicates that the spatial variation of structural parameters, rather than a particularly long heminode, is the mechanism promoting precisely timed depolarization of the calyx.

The variation of the morphological parameters of myelinated axons that we report here thus plays a crucial role in the processing of temporal information for sound localization (as does the presence of myelin itself^44^,^45^), although we cannot rule out an additional influence of factors we have not investigated, such as differences in ion channel density. More generally, given that remodelling of the properties of myelinated axons can occur in adults^46^, our data highlight the fact that adjustment of parameters such as internode length, internodal axon diameter and node diameter can optimize information processing, and may contribute to the changes of white matter structure that contribute to learning^47^.

## Methods

All animal procedures were performed in accordance with the German guidelines for the care and use of laboratory animals as approved by the Regierung of Oberbayern (AZ 55.2-1-54-2531-105-10, Bavaria, Germany). Mongolian gerbils (*Meriones unguiculatus*) were housed in a vivarium with a normal light–dark cycle (12 h light/12 h dark). Gerbils of both sexes, aged P25–30, were used for the anatomical experiments (*in vitro* axon tracing, immunohistochemistry and electron microscopy).

### *In vitro* axon tracing

Gerbils were deeply anaesthetized with pentobarbital (2 mg per kg body weight) and intracardially perfused with ice-cold Ringer's solution containing heparin. After decapitation, the brainstem was quickly removed from the skull under ice-cold dissecting artificial cerebrospinal fluid (aCSF) comprising (in mM) 125 NaCl, 2.5 KCl, 1 MgCl_2_, 0.1 CaCl_2_, 25 glucose, 1.25 NaH_2_PO_4_, 25 NaHCO_3_, 0.4 ascorbic acid, 3 myoinositol and 2 pyruvic acid (all chemicals from Sigma-Aldrich). For anterograde tracing of GBC axons, borosilicate micropipettes with a tip diameter of 10–15 μm were filled with a 10% solution of tetramethylrhodamine dextran (3,000 molecular weight (MW); Invitrogen, La Jolla, CA) and visually guided to the anterior ventral cochlear nucleus (AVCN) in brainstem explants using a stereo microscope. Tracer was pressure-injected (15 pounds per square inch (PSI)) into the GBC area of the AVCN using a picospritzer (Picospritzer III, Parker, Cleveland, OH), followed by several electroporation pulse trains (modified from another study^40^). Pulses (50 ms) had an amplitude of 50 V and were applied at 10 Hz using an isolated pulse stimulator (A-M Systems). For anterograde tracing of SBC axons the brainstem was sectioned along the posterior–anterior axis until the outlines of the LSO were clearly visible. Tracer was applied in the region of the medial limb of the LSO through pressure injections and electroporation as described above. SBC fibres (originating in the cochlear nucleus) pass through this region on their way to the contralateral MSO. This allowed us to label SBC axons without labelling GBC axons, and to reduce the time needed for the tracer to diffuse within the axons to the contralateral MSO. Subsequently, the explants were transferred to a chamber with oxygenated incubating aCSF (the same as the dissecting aCSF described above except with 2 mM instead of 0.1 mM CaCl_2_), and incubated for 2 h at room temperature to allow for homogeneous distribution of the tracer in axons. Thereafter, the brainstems were immersion-fixed at room temperature overnight in 4% paraformaldehyde (PFA) solution.

### Immunohistochemistry

Brainstems were sectioned coronally at 120 μm using a vibratome slicer. After rinsing in PBS, sections were transferred to a blocking solution containing 1% bovine serum albumin, 2% Triton X-100 and 0.1% saponin in PBS. Multiple-immunofluorescence labelling was performed with the following primary antibodies (incubation time 2 days): ankyrin G (Santa Cruz, USA; sc-28561; rabbit polyclonal IgG; 1:500); K_v_1.2 (NeuroMab, USA; 75-008 clone K14/16; Mouse IgG2b; 1:500); NFH (AbCam, UK; ab4680; chicken polyclonal IgY; 1:400); Pan Na_v_ (Sigma, Germany; S6936; rabbit polyclonal IgG; 1:300); MAP2 (Neuromics, USA; CH22103 chicken polyclonal, 1:1,000). After incubation with secondary antibodies overnight, sections were rinsed 3 × 10 min in PBS and coverslipped with Vectashield mounting medium.

### Confocal microscopy

Confocal optical sections were acquired with a Leica TCS SP5-2 confocal laser-scanning microscope (Leica Microsystems, Mannheim, Germany) equipped with HCX PL APO CS 20X/NA0.7, HCX PL APO Lambda Blue × 63/numerical aperture 1.4 immersion oil and HCX PL APO × 63/numerical aperture1.3 glycerol 37 °C objectives. Fluorochromes were visualized with excitation wavelengths of 405 nm (emission filter 410–430 nm) for aminomethylcoumarin (AMCA), 488 nm (emission filter 510–540 nm) for Alexa 488 and DyLight 488, 561 nm (emission filter 565–585 nm) for Cy3 and tetramethylrhodamine dextran, 594 nm (emission filter 605–625 nm) for Texas Red or DyLight 594 and 633 nm (emission filter 640–760 nm) for Nissl Deep Red and Cy5. For each optical section the images were collected sequentially for two to five fluorochromes. Stacks of 8-bit greyscale images were obtained with axial distances of 290 nm between optical sections and pixel sizes of 120–1,520 nm depending on the selected zoom factor and objective. To improve the signal-to-noise ratio, images were averaged from four successive scans. After stack acquisition, chromatic aberration-induced Z shift between colour channels was corrected for using a custom plugin written by Dr Boris Joffe. RGB stacks, montages of RGB optical sections and maximum-intensity projections were assembled using the ImageJ 1.37k plugins and Adobe Photoshop 8.0.1 (Adobe Systems, San Jose, CA) software. For the morphometric analysis of traced GBC and SBC fibres, overlapping stacks of images were acquired from coronal brainstem slices (120 μm thick) at the level of the trapezoid body in the area spanning the MNTB and the contralateral tracer injection site (cochlear nucleus; voxel size: 482 × 482 × 290 nm) and in the area between MSO and the contralateral injection site (medial limb of the LSO; voxel size: 241 × 241 × 290 nm), respectively.

### Identification of GBC and SBC axons

GBC fibres were unambiguously identified as such from their prominent giant presynaptic endings (calyces of Held) that terminated in the contralateral MNTB. On the basis of their exact site of termination in the MNTB (in the medial or lateral half of the MNTB), GBC axons were classified as being either medially or laterally terminating. MNTB borders were determined using a counterstain (MAP2 or fluorescent Nissl stain). SBC fibres were identified as axons that could be followed to the medial somatodendritic region of the MSO (the MSO contralateral to where tracer was applied in the medial limb of the LSO; see also section on *in vitro* axon tracing above). Some fibres formed varicosities in this region. Other fibres became very thin in the medial dendritic region and could not be followed any further, indicating that they terminated there. Since they were located in the middle of the slice (not on the surface), we can rule out that they were cut during the slicing procedure.

### Morphometry

Using the ImageJ 1.37k paint-brush tool, individual axons of GBCs and SBCs filled with tetrametylrhodamine dextran were manually labelled by following a single axon successively through each optical section of the confocal image stack. Subsequently, the neighbouring axons were digitally deleted. We refer to this method as digital extraction. The same axon was identified in the neighbouring overlapping confocal image stacks and digitally extracted. This allowed us to follow single axons for up to ∼2,200 μm. Nodes of Ranvier were identified on the basis of immunohistochemical labelling of nodal and juxtaparanodal marker proteins (Ankyrin G and K_v_1.2). Distances between nodes of Ranvier (that is, internode lengths measured mid-node to mid-node) were measured in three dimensions in confocal image stacks using the ImageJ 1.37k Sync Measure three-dimensional tool. Internodal axon diameter and node diameter measurements were made in two dimensions from maximum-intensity projections of confocal image stacks.

### Electron microscopy

Gerbils were deeply anaesthetized with pentobarbital (2 mg per kg body weight) and intracardially perfused with Ringer's solution. This was followed by perfusion with 2.5% glutaraldehyde plus 2% PFA in cacodylate buffer (CB). Subsequently, the brainstem was removed from the skull and postfixed in the same fixative overnight at 4 °C. After washing for 3 × 10 min in CB, brainstems were sectioned parasagittally at 200 μm using a vibratome slicer. Thereafter, a 1 mm × 1 mm block containing the trapezoid body fibres was extracted using a razor blade. Tissue was then washed four times in CB and postfixed in 1% OsO_4_ in CB for 1–2 h. After washing and dehydrating in graded series of acetone, tissue was embedded in resin. Before ultrathin sectioning, several semithin sections were cut for light microscopic investigation.

### Statistics

Data are represented as mean±s.e.m. Tests for normal distribution and equal variance were performed using the Kolmogorov–Smirnov test and the Levene Median test, respectively. *P* values are from Student's two-tailed *t*-tests (for normally distributed data) or Mann–Whitney rank sum tests (for data that were not normally distributed). Tests were performed using the SigmaStat 3.5 software. For multiple comparisons, *P* values were corrected using a procedure equivalent to the Holm–Bonferroni method (for *N* comparisons, the most significant *P* value is multiplied by *N*, the second most significant by *N*−1, the third most significant by *N*−2 and so on; corrected *P* values are significant if they are less than 0.05). Data presented in Fig. 1d and Fig. 2c,e,g,i were fit with linear or exponential functions using the Igor Pro 5.02 (Wavemetrics).

### Simulations

The myelinated axon model of Halter and Clark^48^ was implemented in MATLAB, modified to remove the periaxonal space in that model by setting its conductivity to zero and adapted to the dimensions of GBClat, GBCmed and SBC axons. The values for the geometrical parameters are shown in Supplementary Tables 1–3, and the values for the electrophysiological parameters are shown in Supplementary Table 4. Keeping the electrophysiological parameters fixed for all the simulations allows us to demonstrate that the differences of anatomical parameters alone suffice to determine the relative conduction speed of the different axons and to determine whether or not APs propagate reliably into the calyx.

The *trans*-myelin capacitance was calculated according to the equation *C*_my_=2*πLc*_my_*/*(Σ(1*/r*_*i*_)), where the *r*_*i*_ are the radii of the individual myelin membranes making up the sheath (assumed to occur at equally spaced radii between the axon radius and the outer radius of the sheath), *L* is the internode length and *c*_my_ is the specific capacitance/*m*^2^ of a single membrane in the myelin. The *trans*-myelin conductance was calculated in a similar way. The differential equations of the model were derived and solved according to the method used in ref. 48. For simulations examining the conduction velocity far from the calyx, 200 internodes were simulated with each internode being represented as 10 segments, an AP was initiated in the first node through brief current stimulation and the conduction speed was derived from the time needed for the AP to pass from the 71st to the 90th internode. The nodes of Ranvier contained inactivating Na^+^ and low-threshold K^+^ (K_LT_) channels with Hodgkin–Huxley-like kinetics taken from refs 49, 50, respectively, as well as a leak conductance. Explicitly, the equation governing current flow across the membrane in the node was:


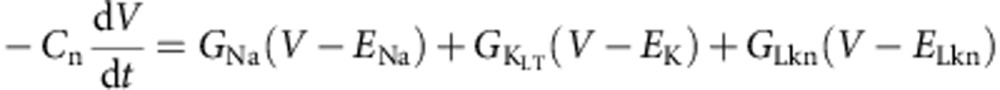


where *C*_n_ is the capacitance of the node (*C*_n_=*πL*_n_*d*_n_*c*_n_, where *L*_n_ is the length of the node, *d*_n_ is the diameter of the node and *c*_n_ is the specific capacitance of the nodal membrane), *G*_Lkn_ is the leak conductance in the node (*G*_Lkn_=*πL*_n_*d*_n_*g*_Lkn_, where *g*_Lkn_ is the specific leak conductance of the nodal membrane) and *V* is the (time-varying) membrane potential in the node. *E*_Na_, *E*_K_ and *E*_Lkn_ are the Na^+^, K^+^ and nodal leak reversal potentials, respectively, and


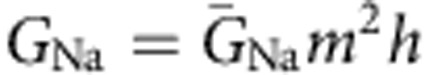



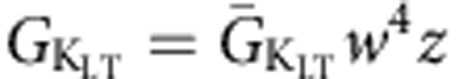


where 
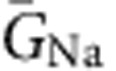
 and 
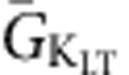
 are the peak conductances of the Na^+^ and K^+^ currents, respectively, in the node (derived from the specific conductances in a similar manner to *G*_Lkn_). The variables *m*, *h*, *w* and *z* are functions of membrane potential and time, and satisfy:


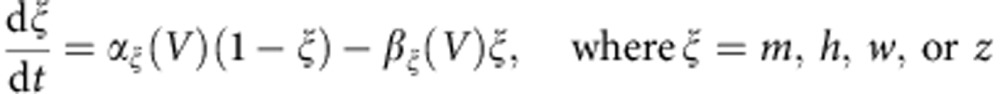


and


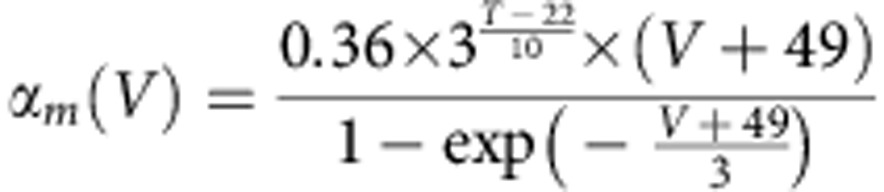



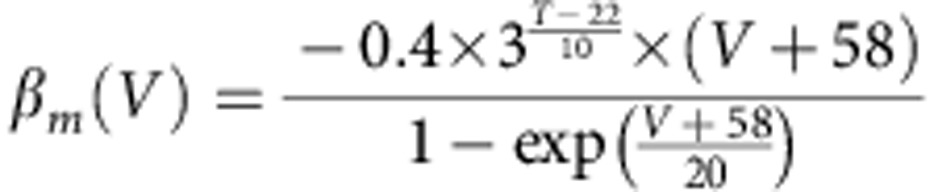



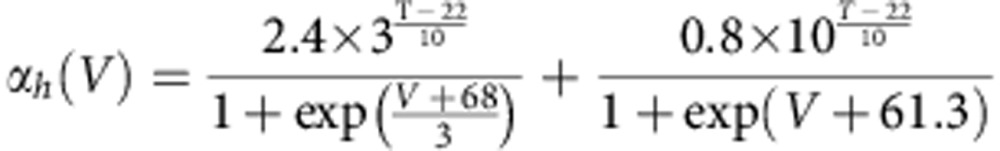



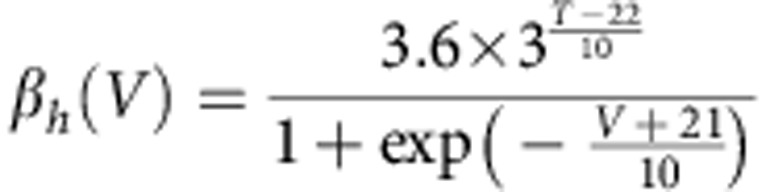



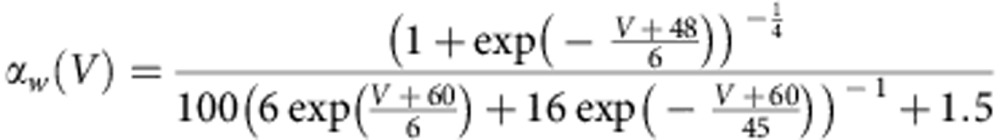



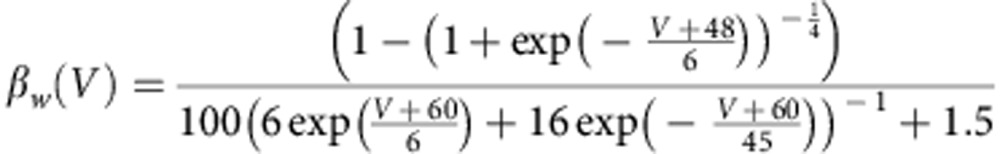



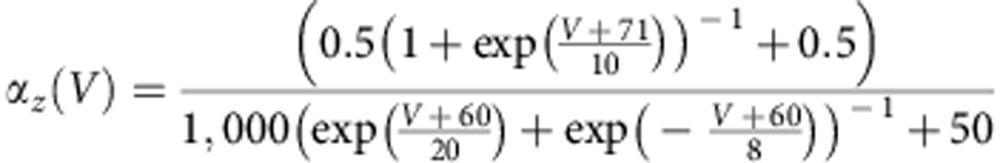



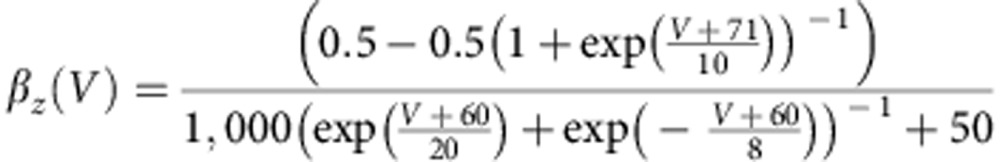


where, for all these equations, *V* is in mV and the rate constants are in ms^−1^. The Na^+^ current kinetics were adjusted for temperature, *T* (in °C), as written in the equations above. The low-threshold K^+^ current kinetics were defined at 22 °C and similarly adjusted for temperature using a *Q*_10_ value of 3.0.

For simulations examining AP propagation into the calyx of Held, 50 internodes were simulated and APs were again initiated in the first node of Ranvier. The end terminal was simulated by extending the last node by three compartments corresponding to the heminode, post-heminode (represented as 10 segments) and calyx. The morphometric parameters for the heminode and post-heminode were taken from this paper (Supplementary Table 3). The heminode contained the inactivating Na^+^, low-threshold K^+^ and leak channels as described above for the nodes of Ranvier. The post-heminode was taken to be a capacitor without conductance. The calyx was represented as a single cylinder (divided into 50 equal serial segments), equivalent to five smaller cylinders representing the main processes of the calyx structure, with a surface area of 1,700 or 1,250 μm^2^ (see main text), consistent with published values^39^. Membrane potential was measured in the most distal segment of the calyx. When investigating the effect of altering calyx geometry, the radius of these smaller cylinders was varied, with corresponding alterations in their length to keep the surface area constant. A single cylinder of *A *μm diameter and *L *μm length can be shown to be equivalent to *N* parallel cylinder ‘processes' each with diameter *A*/*N*^2/3 ^μm and length *L*/*N*^1/3 ^μm. Therefore, a simulated calyx equivalent cylinder of 10 μm diameter and 54.1 μm length (giving a surface area of 1,700 μm^2^) represents five parallel cylinders of diameter 3.42 μm and length 31.6 μm. The calyx contained high-threshold K^+^ (*K*_HT_) and hyperpolarization-activated cation (*I*_h_) conductances with Hodgkin–Huxley-like kinetics taken from ref. 50. It also contained a calcium conductance taken from ref. 51. Explicitly, the equation governing current flow across the membrane in the calyx was:


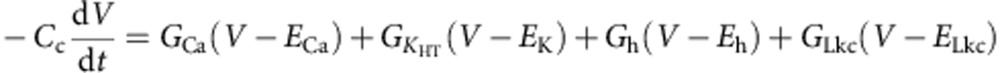


where *C*_c_ is the capacitance of the calyx, *G*_Lkc_ is the leak conductance in the calyx, *E*_Ca_ and *E*_h_ are the reversal potentials of the calcium and *I*_h_ currents and,


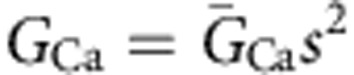



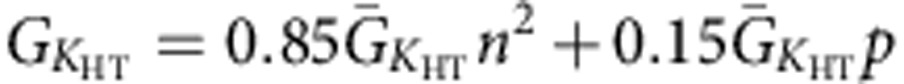



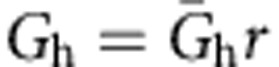


where 
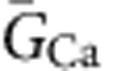
, 
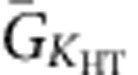
 and 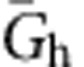 are the peak conductances (conductance values were taken from ref. 52, but 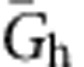 was reduced 10-fold to increase the input resistance of the calyx to make it more similar to published values (Forsythe measured a value of 388±228 MΩ (ref. 53); in the model, GBClat and GBCmed calyces had input resistances of 240 and 325 MΩ, respectively), and *s*, *n*, *p* and *r* are functions of membrane potential and time that satisfy:


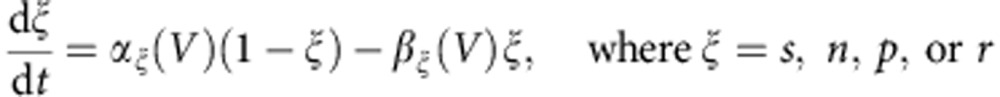


and


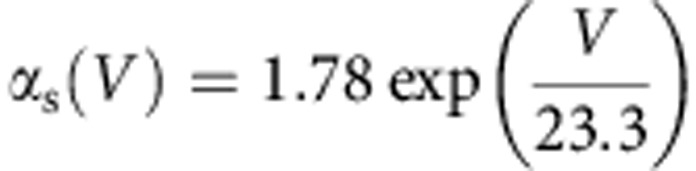



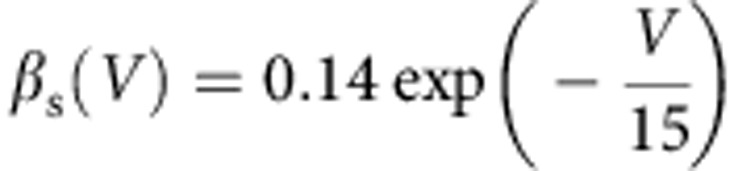



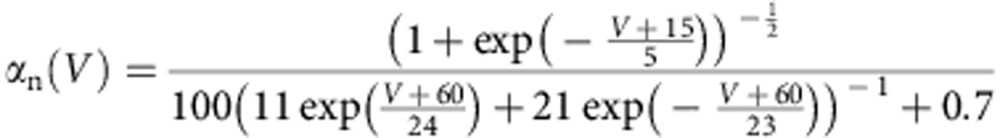



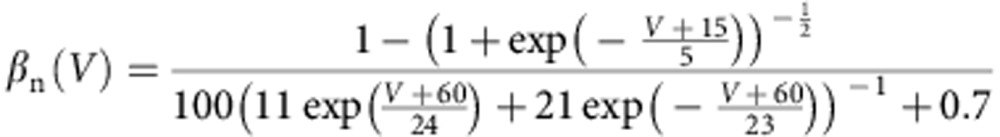



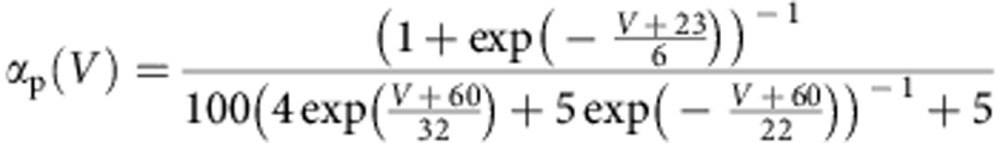



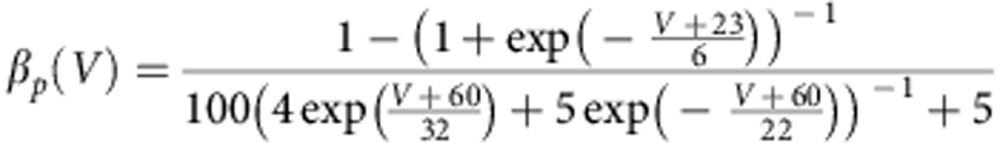



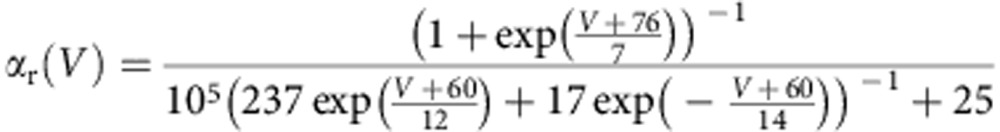



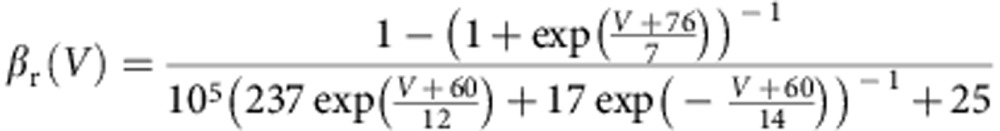


For all of these equations *V* is in mV and the rate constants are in ms^−1^. The Ca^2+^ current kinetics were defined at 24 °C, and the high-threshold K^+^ and *I*_h_ current kinetics were defined at 22 °C. All were adjusted for temperature with a *Q*_10_ value of 3.0. The computer code will be made available on request.

### *In vitro* electrophysiology

All experimental procedures were approved by the Bavarian district government and performed according to the European Communities Council Directive (2010/63/EU). Mongolian gerbils (*M. unguiculatus*) of either sex (P17–P29, *n*=14) were killed by decapitation. Coronal brainstem sections (200–300 μm thick) containing the superior olivary complex (SOC) were cut in a high-sucrose, low-sodium aCSF at room temperature (∼24 °C). After slicing, the brainstem slices were maintained in a normal ACSF at 37 °C for 30–45 min, after which they were stored in a slice-maintenance chamber at room temperature (∼24 °C). Composition of the normal ACSF in mM: NaCl 125, KCl 2.5, NaHCO_3_ 26, glucose 10, NaH_2_PO_4_ 1.25, sodium pyruvate 2, myoinositol 3, CaCl_2_ 2, MgCl_2_ 1 and ascorbic acid 0.5. pH was 7.4, bubbled with 95% O_2_, 5% CO_2_. For the low-sodium ACSF, CaCl_2_ and MgCl_2_ concentrations were 0.1 and 4 mM, respectively, and NaCl was replaced by 200 mM sucrose. Experiments were conducted at 36±1 °C, maintained by an inline feedback temperature controller and heated stage (TC344B, Warner Instruments, Hamden, CT, USA) with the recording chamber being continuously perfused with ACSF at a rate of 1–2 ml min^−1^. Whole-cell patch-clamp recordings were made from visually identified MNTB neurons (OlympusBX51WI microscope) using an EPC 10/2HEKA amplifier, sampling at 20–50 kHz and filtering at 10 kHz. Patch pipettes were pulled from borosilicate glass capillaries (GC150F-7.5, optical density: 1.5 mm; Harvard Apparatus, Edenbridge, UK) using a DMZ Universal puller (Zeitz), filled with a patch solution containing (in mM): K-gluconate 97.5, KCl 32.5, HEPES 40, EGTA 5, MgCl_2_ 1, Na_2_ phosphocreatine 5 (all chemicals from Sigma-Aldrich). pH was adjusted to 7.2 with KOH. Electrode resistance was between 2.4 and 6.8 MΩ. Synaptic potentials were evoked by afferent fibre stimulation with two concentric bipolar electrodes (FHC; Bowdoin, ME, USA), one placed near the midline over the trapezoid body fibres and the other one further on the contralateral side, driven by voltage pulses generated by the HEKA amplifier and post-amplified by a linear stimulus isolator (Pulse Stimulator AM-2100). Latencies were measured from the onset of the stimulus artefact to the half-maximum of the postsynaptic response. Only EPSCs greater than 2 nA were considered calyceal and used for analysis. The distance between the tip of the recording electrode and the midline of the slice, and the distance between the tip of the recording electrode and the stimulation electrodes, were measured using ImageJ.

### *In vivo* electrophysiology

All experiments were performed in accordance with the German guidelines for the care and use of laboratory animals as approved by the Regierung of Oberbayern (AZ 55.2-1-54.2531.8-211-10). Twelve adult Mongolian gerbils (*M. unguiculatus*) of either sex were initially anaesthetized with an intraperitoneal injection (0.5 ml per 100 g body weight) of a mixture of ketamine (20%) and xylazin (2%) diluted in 0.9% NaCl solution. During surgery and recording, animals were injected continuously with the same anaesthesia via an automatic pump (801 Syringe Pump, Univentor, Malta) at a pump rate of 1.6–2.5 μl min^−1^ depending on body weight. The animal was transferred to a sound-attenuated chamber and mounted in a custom-made stereotactic instrument^54^. Constant body temperature was maintained at 37 °C using a thermostatically controlled heating blanket. To gain access to the MNTB and AVCN, two craniotomies were performed: 700–1,200 μm lateral to the midline above the lambda suture and 3,000–3,600 μm lateral to the midline caudal to the posterior aspect of the transverse sinus. After recording the animal was killed with a lethal dose of pentobarbital (20 mg ml^−1^).

Single-unit responses of neurons in the MNTB elicited by acoustic and electric stimulations were recorded extracellularly with glass electrodes (3–37 MΩ) filled with 3 M KCl. The recording electrode was tilted rostrally by 7° and advanced under remote control, using a micromanipulator (IVM-1000, Scientifica, UK). The lateral position of the electrode was determined with regard to lambda, which was taken as a reference point and corresponds to the location of the recorded neuron within the MNTB. Recordings were preamplified (Patch clamp EPC 10 USB, Heka, Germany), filtered (Humbug 50/60 Hz noise eliminator, Quest Scientific, USA) and fed to a computer via a Multi I/O processor (RZ6, Tucker-Davies Technology, Alachua, FL). The voltage traces were digitized at a sampling rate of 48 kHz and band-pass-filtered (0.4–6 kHz). Isolation of APs from single neurons was guaranteed by visual inspection of amplitude and shape during the experiment and by off-line spike-cluster analysis (Brainware, Tucker-Davies Technology). Only neurons with characteristic complex waveforms, consisting of a prepotential, which reflects the activity of the presynaptic calyx of Held terminal, followed by a postsynaptic spike, were included.

Acoustic stimuli were digitally generated at a sampling rate of 200 kHz by TDT System III (Tucker-Davies Technology), digitally attenuated and converted to analogue signals (RZ6, Tucker-Davies Technology), and then delivered to earphones (ER 4PT, Etymotic Research, Elk Grove Village, IL). Monaural stimulation with pure tones (100 ms duration plus 5 ms cosine rise/fall time) of predefined frequency/intensity combinations (six intensities ranging from 5 dB below to 45 dB above threshold in 10 dB steps and nine frequencies ranging from 0.2*CF to 1.8*CF in steps of 0.2*CF) were used to determine the CF of the MNTB neuron, which is the frequency that elicits spikes at the lowest sound intensity.

Electric stimulation was performed using a 16-channel tungsten electrode microwire array (Omnetics-based electrodes, Tucker-Davies Technology) that was inserted into the AVCN, contralateral to the MNTB that was recorded from. The electrode array was tilted caudally by 13° and advanced under remote control, using a micromanipulator (IVM-1000, Scientifica). After encountering multiunit auditory activity in the AVCN (RA16PA 16-channel medusa preamplifier and RX5-2 pentusa base station, Tucker-Davies Technology), GBCs were electrically stimulated using one channel at a time. Electric stimuli were biphasic cathodic leading current pulses (120 μs per phase, 40 μs interphase gap, 2 Hz repeat rate, 0–40 μA amplitude in 2 μA steps), that were digitally generated at a sampling frequency of 25 kHz and passed to the electrode channels (RX7-2 stimulator base station and MS16 stimulus isolator ,Tucker-Davies Technology).

The precise measurement of the time period between the peak of the stimulation artefact and the peak of the prepotential for the calculation of the axonal AP propagation latency was performed by custom-made programmes in Matlab (The MathWorks). The latency was determined at the current amplitude that elicited spikes in at least 50% of the trials (that is, minimal stimulation close to threshold) and is shown as a mean value.

## Additional information

**How to cite this article:** Ford, M. C. *et al.* Tuning of Ranvier node and internode properties in myelinated axons to adjust action potential timing. *Nat. Commun.* 6:8073 doi: 10.1038/ncomms9073 (2015).

## Supplementary Material

Supplementary Information Supplementary Figure 1, Supplementary Tables 1-4 and Supplementary References
