# Supplementary material for: Tuning of Ranvier node and internode properties in myelinated axons to adjust action potential timing
Source: Nat Commun. 2015 Aug 25;6:8073. doi: 10.1038/ncomms9073 (PMC4560803; doi:10.1038/ncomms9073)
Supplement: Supplementary Information — Supplementary Figure 1, Supplementary Tables 1-4 and Supplementary References [file ncomms9073-s1.pdf]

Supplementary Information

Tuning Ranvier node and internode properties in myelinated axons to adjust action potential timing

Marc C. Ford, Olga Alexandrova, Lee Cossell, Annette Stange –Marten, James Sinclair, Conny Kopp-Scheinpflug, Michael Pecka, David Attwell and Benedikt Grothe

Supplementary figure 1

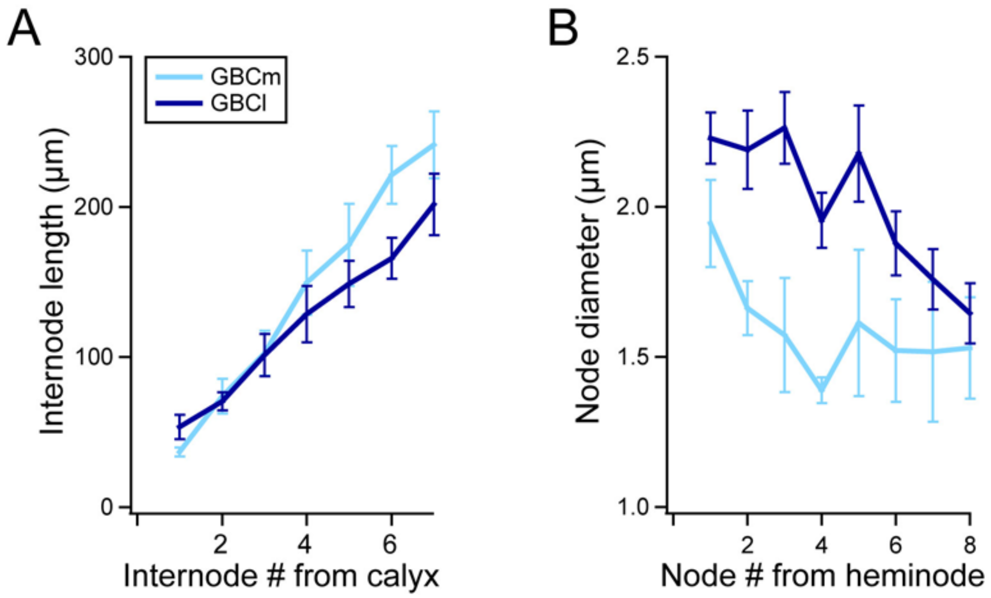

Supplementary Figure 1 Internode lengths and node diameters are graded in the distal part of GBC fibres.

(**A**) In GBCmed fibres the mean internode length decreases progressively from 242±22 µm (7th internode from calyx) to 36.9±3.0 µm (last internode; p<0.001; Mann-Whitney rank sum test). A comparable decrease in mean internode length from 202±20 µm (7th internode from calyx) to 53.5±8.3 µm (last internode) was found in GBClat fibers (p<0.001; Student's t-test). (**B**) In GBCmed fibers the mean diameter of nodes of Ranvier increases from 1.39±0.04 µm (4th node from calyx) to 1.95±0.15 µm (last node; p=0.002). A similar increase in node of Ranvier mean diameter from 1.65±0.10 µm (8th node from calyx) to 2.23±0.09 µm (last node) was found in GBClat fibers (p<0.001).

Supplementary Table 1. Geometrical parameters of GBClat, GBCmed and SBC axons

along the part of the axon distant from the calyx

Parameter	Value	Unit
Internode axon diameter		
GBC lateral	3.06	µm
GBC medial	2.41	µm
SBC	1.35	µm
Internodal myelin diameter		
GBC lateral	4.01	µm
GBC medial	3.20	µm
SBC	1.77	µm
Node diameter		
GBC lateral	1.68	µm
GBC medial	1.50	µm
SBC	0.85	µm
Internode length		
GBC lateral	197.7	µm
GBC medial	238.6	µm
SBC	163.8	µm
Node length	1	µm
Number of myelin layers		
GBC lateral	31	
GBC medial	26	
SBC	14	

Supplementary Table 2. Graded geometrical parameters of GBC axons near calyx

Parameter	GBClat value	GBCmed value	Unit
Internode length			
1st (internode from calyx)	53.5	36.9	µm
2nd	70.7	73.7	µm
3rd	101.5	102.4	µm
4th	128.5	149.5	µm
5th	148.6	174.7	µm
6th	166.0	221.2	µm
Internode axon diameter			
1st (internode from calyx)	3.44	2.38	µm
2nd	3.62	2.47	µm
3rd	3.34	2.31	µm
4th	3.17	2.38	µm
5th	3.28	2.19	µm
6th	3.22	2.53	µm
Node diameter			
1st (node from calyx)	2.23	1.95	µm
2nd	2.19	1.66	µm
3rd	2.26	1.57	µm
4th	1.95	1.39	µm
5th	2.18	1.61	µm
6th	1.88	1.52	µm

Supplementary Table 3. Geometrical parameters of GBC axon terminals

Parameter	GBClat value	GBCmed value	Unit
Heminode			
Diameter	2.04	1.60	µm
Length	2.13	2.18	µm
Postheminode			
Diameter	2.70	2.26	µm
Length	13.22	10.71	µm

Supplementary Table 4. Electrophysiological parameters of the model

Parameter	Explanation	Value	Unit	Source
T	Temperature	37	˚C	
g_{Na}	Nodal peak Na^+ conductance	588	mS/cm^2	1
g_{KLT}	Nodal peak low-threshold K^+ conductance	40	mS/cm^2	2
g_{Lkn}	Nodal leak conductance	1.76	mS/cm^2	Adjusted to set V_{rest}
g_{Ca}	Calyx peak Ca^{2+} conductance	3	mS/cm^2	2
g_{KHT}	Calyx peak high-threshold K^+ conductance	20	mS/cm^2	2
g_h	Calyx peak I_h conductance	0.095	mS/cm^2	2†
g_{Lkc}	Calyx leak conductance	0.094	mS/cm^2	Adjusted to set V_{rest}
g_{Lki}	Internode axon leak conductance	0.1	mS/cm^2	3
g_{my}	Myelin membrane conductance	1	mS/cm^2	3
c	Membrane capacitance (includes node, c_n, internode axon, c_i, myelin, c_{my}, and calyx, c_c, membrane)	1	$\mu F/cm^2$	4
ρ_{ax}	Axoplasmic resistivity	70	Ω cm	3
V_{rest}	Resting membrane potential	−75	mV	5
E_{Lkn}	Nodal leak reversal potential	−72	mV	Adjusted to set V_{rest}
E_{Lkc}	Calyx leak reversal potential	−90	mV	Set to E_K
E_{Lki}	Internode axon leak reversal potential	−75	mV	Set to V_{rest}
E_{Na}	Na^+ reversal potential	+55	mV	6
E_K	K^+ reversal potential	−90	mV	7
E_{Ca}	Ca^{2+} reversal potential	+43.5	mV	8
E_h	I_h reversal potential	−43	mV	9

† Reduced 10-fold to increase the input resistance of the calyx.

Supplementary references

1. Lorincz, A. & Nusser, Z. Molecular identity of dendritic voltage-gated sodium channels. *Science* **328,** 906–909 (2010).
2. Spirou, G. A., Chirila, F. V., von Gersdorff, H. & Manis, P. B. Heterogeneous Ca2+ influx along the adult calyx of held: a structural and computational study. *Neuroscience* **154,** 171–185 (2008).
3. Richardson, A. G., McIntyre, C. C. & Grill, W. M. Modelling the effects of electric fields on nerve fibres: influence of the myelin sheath. *Med. Biol. Eng. Comput.* **38,** 438–446 (2000).
4. Hodgkin, A. L. & Huxley, A. F. A quantitative description of membrane current and its application to conduction and excitation in nerve. *J. Physiol.* **117,** 500–544 (1952).
5. Huang, H. & Trussell, L. O. KCNQ5 channels control resting properties and release probability of a synapse. *Nat. Neurosci.* **14,** 840–847 (2011).
6. Rothman, J. S., Young, E. D. & Manis, P. B. Convergence of auditory nerve fibers onto bushy cells in the ventral cochlear nucleus: implications of a computational model. *J. Neurophysiol.* **70,** 2562–2583 (1993).
7. McIntyre, C. C., Richardson, A. G. & Grill, W. M. Modeling the excitability of mammalian nerve fibers: influence of afterpotentials on the recovery cycle. *J. Neurophysiol.* **87,** 995–1006 (2002).
8. Borst, J. G. & Sakmann, B. Calcium current during a single action potential in a large presynaptic terminal of the rat brainstem. *J. Physiol.* **506 (Pt 1),** 143–157 (1998).
9. Rothman, J. S. & Manis, P. B. The Roles Potassium Currents Play in Regulating the Electrical Activity of Ventral Cochlear Nucleus Neurons. *J. Neurophysiol.* **89,** 3097–3113 (2003).
